# Outcomes of Third-Generation Cephalosporin Plus Ciprofloxacin or Doxycycline Therapy in Patients with *Vibrio vulnificus* Septicemia: A Propensity Score-Matched Analysis

**DOI:** 10.1371/journal.pntd.0007478

**Published:** 2019-06-12

**Authors:** Seong Eun Kim, Sung Un Shin, Tae Hoon Oh, Uh Jin Kim, Kalifa Sanneh Darboe, Seung-Ji Kang, Hee-Chang Jang, Sook-In Jung, Hee-Young Shin, Kyung-Hwa Park

**Affiliations:** 1 Department of Infectious Diseases, Chonnam National University Hospital, Gwang-ju, Republic of Korea; 2 Department of Biomedical Science, Chonnam National University Medical School, Gwang-ju, Republic of Korea; 3 Department of Infectious Diseases, Chonnam National University Medical School, Gwang-ju, Republic of Korea; Lowell General Hospital, UNITED STATES

## Abstract

**Background:**

Combination therapy with a third-generation cephalosporin (TGC) and a tetracycline analogue is recommended for *Vibrio vulnificus* infection. The combination of a TGC and ciprofloxacin has synergistic *in vitro* bactericidal activity against *V*. *vulnificus*. No clinical study has compared the standard regimen with TGC plus ciprofloxacin therapy for *V*. *vulnificus* infection.

**Methods:**

Patients with a confirmed *V*. *vulnificus* infection at two medical centers in Korea from 1991 to 2016 were enrolled in this study. The patients were grouped according to the type of antibiotic administered. A retrospective propensity-score-matched case-control study of patients treated with TGC plus doxycycline or TGC plus ciprofloxacin was performed. The clinical characteristics and outcomes of the patients were analyzed.

**Results:**

A total of 218 patients were confirmed to have *V*. *vulnificus* septicemia during the study, and the 30-day survival rate was 39% (85/218). The patients were classified into the following six treatment groups: TGC monotherapy (n = 82), TGC plus doxycycline therapy (n = 42), TGC plus ciprofloxacin therapy (n = 39), ciprofloxacin monotherapy (n = 14), other β-lactam monotherapy (n = 10), and other (n = 31). The survival rates of these groups were as follows: TGC monotherapy (35%), TGC plus doxycycline (38%), TGC plus ciprofloxacin (54%), ciprofloxacin monotherapy (29%), other β-lactam (20%), and other (39%). The 30-day survival rate showed no significant difference between the TGC plus doxycycline and TGC plus ciprofloxacin groups (log-rank test, *P* = 0.18). Among the 81 patients treated with TGC plus doxycycline or TGC plus ciprofloxacin, 12 per treatment group were selected by propensity-score matching. There was no significant difference in the baseline characteristics or the frequency of fasciotomy between the two groups. The 30-day survival rate showed no significant difference between the TGC plus doxycycline (50%) and TGC plus ciprofloxacin (67%) groups (log-rank test, *P* = 0.46).

**Conclusion:**

Our data suggest that the outcome of TGC plus ciprofloxacin therapy was comparable to that of TGC plus doxycycline therapy in patients with *V*. *vulnificus* septicemia.

## Author summary

The combination of a third-generation cephalosporin (TGC) and ciprofloxacin has synergy *in vitro* bactericidal activity against *V*. *vulnificus*. No clinical study has compared the standard regimen with TGC plus ciprofloxacin therapy for *V*. *vulnificus* infection. A total of 218 patients were enrolled who are confirmed to have *V*. *vulnificus* septicemia in two medical centers in Korea from 1991 to 2016. The 30-day survival rate was 39% (85/218) for all patients, 38% (16/42) for TGC plus doxycycline and 54% (21/39) for TGC plus ciprofloxacin (log rank test, *P* = 0.18). A propensity score-matched analysis was performed and 12 per treatment groups were selected. The 30-day survival rate showed no significant difference between the TGC plus doxycycline (50%, 6/12) and TGC plus ciprofloxacin (67%, 4/12) groups (log-rank test, *P* = 0.46). The outcome of TGC plus ciprofloxacin therapy was comparable to that of TGC plus doxycycline therapy in patients with *V*. *vulnificus* septicemia.

## Introduction

*Vibrio vulnificus* is a Gram-negative halophile that thrives in warm marine and estuarine environments worldwide [1]. *V*. *vulnificus* produces toxins and enzymes, including capsular polysaccharides, metalloproteases, lipopolysaccharides, and cytolysin [2, 3]. These toxins and enzymes cause extensive tissue damage and play a major role in the development of sepsis. *V*. *vulnificus* causes foodborne disease and wound infections. Primary septicemia caused by *V*. *vulnificus* is characterized by bacteremia without any obvious focus of infection and usually presents with a sudden onset of fever and chills, often accompanied by vomiting, diarrhea, abdominal pain, and pain in the extremities within 7 days after the ingestion of contaminated seafood. Within the first 24 h after the onset of illness, secondary cutaneous lesions such as cellulitis, ecchymosis proceeding to hemorrhagic bullae begin to appear on the extremities [4, 5]. This primary septicemia is the most lethal infection caused by *V*. *vulnificus*, with an average mortality rate exceeding 50% [6–8]. In addition to septicemia, serious wound infection can be produced by *V*. *vulnificus* [9]. Like systemic disease, wound infections progress rapidly to cutaneous lesions which can progress to necrotizing fasciitis at the site of infection in patients with the underlying disease. However, mortality rate (about 25%) for wound infections is lower than that of primary septicemia [10, 11].

In patients with necrotizing skin and soft tissue infections (NSTI) caused by *V*. *vulnificus*, early surgical treatment (within 12–24 h of admission) is important to achieve a favorable outcome because the necrotic tissue has an insufficient blood supply to achieve an effective concentration of any antimicrobial agent [12, 13]. The role of antibiotic therapy is to eradicate viable pathogens in the blood or inflamed but still well-perfused tissue and prevent their further spread. Although a variety of antibiotic agents are effective against *V*. *vulnificus in vivo* or *in vitro*, including erythromycin, tetracycline, cephalosporin, tigecycline, ciprofloxacin and extended-spectrum penicillins [14–17], clinical data on antibiotic efficacy are lacking. Two large retrospective clinical studies of antibiotic efficacy showed that a third-generation cephalosporin (TGC) plus tetracycline regimen is optimal for *V*. *vulnificus* infections with necrotizing skin lesions [18, 19]. Indeed, the synergistic effect of TGC plus tetracycline therapy has been demonstrated in mice [20–22]. Based on these studies, the Centers for Disease Control and Prevention (CDC) recommends a TGC in combination with intravenous or oral doxycycline for the management of *V*. *vulnificus* wound infections [23]. Fluoroquinolones have equivalent efficacy to cefotaxime plus minocycline in inhibiting *V*. *vulnificus* in a wound-infection model [24]. Ceftriaxone-ciprofloxacin was reported to be as effective as ceftriaxone-doxycycline in a model of foodborne *V*. *vulnificus* septicemia [22]. We showed that the combination of cefotaxime and ciprofloxacin was more effective in clearing *V*. *vulnificus in vivo* than previously used regimens in a model of subcutaneous wound infection [25].

A study of mortality associated with all generation cephalosporin plus quinolone regimens, including 98 cases from The United States CDC Cholera and Other Vibrio Illness Surveillance (COVIS) dataset from 1990 to 2010 did not find sufficient clinical evidence of the efficacy of TGC plus ciprofloxacin against *V*. *vulnificus* infections [17]. We thus conducted a retrospective study of the clinical efficacy of TGC plus ciprofloxacin in comparison with TGC plus doxycycline by propensity-score matching for the treatment of septicemia and/or NSTI caused by *V*. *vulnificus*. In addition, we evaluated prognostic factors for mortality in patients with a *V*. *vulnificus* infection.

## Materials and methods

### Ethics statement

The analysis of cases was approved by the institutional review board (IRB) of Chonnam National University Hospital and Chonnam National University Hwasun Hospital (IRB No. CNUH-2018-301, CNUHH-2018-180). All analysed data were anonymized.

### Participating hospitals and subjects

From January 1991 to December 2016, patients aged > 18 years diagnosed with a culture-confirmed *V*. *vulnificus* infection and hospitalized at Chonnam National University Hospital (a 1,000-bed tertiary teaching hospital) or Chonnam National University Hwasun Hospital (a 700-bed branch hospital opened in 2004 and tertiary teaching hospital) were enrolled. A systematic review of the patients’ medical records was performed. NSTI caused by *V*. *vulnificus* were diagnosed if both of the following conditions were met: (1) patients with infected skin lesions such as bullae, ecchymosis, or cellulitis and (2) the identification of *V*. *vulnificus* in blood and/or bulla or tissue culture specimens. Primary septicemia was defined as *V*. *vulnificus* bacteremia with no other obvious source of infection. Our hospitals serve Chonnam Province, including the surrounding islands (approximately 3,000), in South Korea. This region has a population of around 3.4 million persons.

### Data collection and definitions

*Vibrio vulnificus* isolates were identified by conventional methods using ID-GNB Vitek 2 cards (bioMérieux, Vitek Inc., Hazelwood, MO, USA). We collected clinical and laboratory information, including age, sex, pre-existing illnesses, site of the NSTI, involved area, symptoms at admission, vital signs, physical and laboratory findings at the time of admission, type of surgical treatment, timing of surgery, antibiotics used, and outcomes. The primary outcomes were the 30-day mortality rate. The severity of illness at admission was evaluated using the first-day Acute Physiology and Chronic Health Evaluation (APACHE) II score. Sepsis and septic shock were defined according to the guidelines of the American College of Chest Physicians and Society for Critical Care Medicine [26]. Enrolled patients received antibiotics as soon as possible within 12 h after admission. Early surgical intervention was defined as fasciotomy or the debridement of necrotic tissue within 24 h of arrival. The early mortality was defined as death within 48 h of admission. The fatal group included patients who died within 30 days of hospital admission. Liver cirrhosis was diagnosed when patients had advanced fibrosis or extrahepatic manifestation of cirrhosis in the imaging findings. Chronic heavy alcohol drinker was defined as 8 or more drinks for women and 15 or more drinks a week for men and one drink equivalent is defined as 14 grams of pure alcohol consumption [27].

### Statistical analysis

Categorical variables were expressed as percentages and continuous variables as means and standard deviations (SD) or medians and interquartile ranges (IQR). In univariate analysis, Pearson’s chi-squared or Fisher’s exact test was used for comparisons of dichotomous variables, and Student’s *t*-test or the Mann-Whitney U test for continuous variables.

Variables of the fatal and nonfatal groups were compared by univariate analysis to identify risk factors for mortality. Next, a multiple logistic regression analysis of factors found to be significant (*P* < 0.20) in the univariate analysis was performed; a value of *P* < 0.05 was regarded as indicative of statistical significance. The examined variables included infection-related factors that had the potential to modulate mortality, in addition to other demographic and therapeutic variables.

A Kaplan-Meier survival analysis and the log-rank test were performed to compare the efficacy of the antibiotic regimens. Propensity-score adjustment was performed to adjust for the following confounding variables: chronic hepatitis B, white blood cell count, shock at admission, early surgical intervention, APACHE II score, heavy alcohol drinking, hemoglobin level, platelet level, and the involvement of two or more extremities (which is associated with the prognosis of *V*. *vulnificus* infection) [18, 28, 29]. This technique permitted the 1:1 pair-matched selection of patients in each antibiotic treatment group. Statistical analyses were performed using SPSS version 24.0 software (SPSS, Inc., Chicago, IL, USA) and GraphPad Prism version 7.0 software (GraphPad Software, La Jolla, CA, USA).

## Results

### Baseline characteristics of the patients with a *V*. *vulnificus* infection

In total, 218 patients with *V*. *vulnificus* septicemia were enrolled in the study. Among them, *V*. *vulnificus* was isolated from the blood of 171 patients, the tissue of 68 patients, and the bullae of 56 patients. Overall, 199 patients (91%, 199/218) had necrotizing skin lesions and 81 patients (37%, 81/218) died within 48 h of arrival; the 30-day mortality rate was 61% (133/218). The demographic, clinical, and laboratory characteristics of the patients are summarized in Table 1. The mean age of the patients was 57.5 years and the majority were males. Chronic hepatitis B (79%), liver cirrhosis (19%), and diabetes mellitus (14%) were the three leading underlying diseases. Almost half of the patients (49%) presented with septic shock at admission. The median APACHE II score on arrival was 13.0. The fatal subgroup had a higher APACHE II score and a higher proportion of septic shock at admission than the nonfatal subgroup. Patients in the nonfatal subgroup underwent early surgical treatment more frequently than those in the fatal subgroup, but the difference was not significant (Table 1). Of the patients, 60 met the criteria for early surgical intervention and 30 received surgical treatment after 24 h (delayed fasciotomy in 10 patients, and necrotic tissue debridement in 20 patients).

**Table 1:** Comparison of demographic, clinical, laboratory findings, and treatment between nonfatal and fatal groups in 218 *V*. *vulnificus* infection patients.

Variables		All patients, n = 218	Nonfatal group, n = 85	Fatal group, n = 133	*P* value §
Age (year), mean ± SD ^b^		57.5 ± 9.6	57.8 ± 10.4	57.4 ± 9.2	0.76
Gender					
	Male ^d^	187 (86)	71 (84)	116 (87)	0.45
	Female	31 (14)	14 (17)	17 (13)	
Accompanied NSTI ^a^^,^ ^d^		199 (91)	79 (93)	120 (90)	0.48
	2 or more extremity involvement ^d^	124/199 (62)	42 (34)	82 (66)	0.08
Raw seafood ingestion history ^d^		180 (83)	70 (82)	110 (83)	0.95
Underlying disease† ^d^					
	Chronic hepatitis B	172 (79)	63 (74)	109 (82)	0.17
	Liver cirrhosis	42 (19)	14 (17)	28 (21)	0.40
	Diabetes mellitus	30 (14)	9 (11)	21 (16)	0.28
	Chronic heavy alcohol drinker	22 (10)	7 (8)	15 (11)	0.47
APACHE II ^a^ score ^c^ (n = 205)		(n = 205)	(n = 79)	(n = 126)	
	Median (IQR)	13 (10, 16.5)	11 (7, 14)	15 (12, 18))	<0.001
WBC ^a^ (×10^9^/L) ^c^		5.3 (2.8, 9.7)	5.3 (3.0, 8.6)	5.4 (2.7, 10.3)	0.79
Hemoglobin (g/L) ^b^		12.2 ± 2.3	12.2 ± 2.2	12.2 ± 2.4	0.82
Platelet (/L) ^c^		53 (33.3, 83.0)	57 (34.5, 84.0)	52 (33, 83)	0.86
Creatinine (mg/dL) ^c^		1.8 (1.2, 2.7)	1.7 (1.3, 2.4)	1.9 (1.2, 2.8)	0.18
Septic shock at admission ^d^		106 (49)	28 (33)	78 (61)	<0.001
Early surgical intervention (within 24 h of arrival) ^d^		60 (28)	28 (33)	32 (24)	0.15
Antibiotics group ^d^					0.31
	TGC monotherapy	82 (38)	29(35)	53(65)	
	TGC-plus-doxycycline	42 (19)	16 (38)	26 (62)	
	TGC-plus-ciprofloxacin	39 (18)	21 (54)	18 (46)	
	Ciprofloxacin monotherapy	14 (6)	4 (29)	10 (71)	
	Other β -lactam	10 (5)	2 (20)	8 (80)	
	Other	31 (14)	12 (39)	19 (61)	

^a^ Abbreviations: NSTI; necrotizing skin and soft tissue infection, APACHE; II acute physiology and chronic health evaluation II, WBC; white blood cells

^b^ Continuous variables are expressed as means ± SD and were compared by the Student t test

^c^ Continuous variables are expressed as medians (IQR) and were compared by the Mann-Whitney U test

^d^ Dichotomous variables were compared by Chi-square test

§ Univariate analysis of fatal and nonfatal group

† One patient might have more than 1 underlying disease

During the study period, various antibiotics were administered. The patients were classified into six treatment groups according to the antibiotic(s) administered (TGC monotherapy, TGC plus doxycycline, TGC plus ciprofloxacin, ciprofloxacin monotherapy, other β-lactam antibiotic, and other [e.g., other combination therapy, aminoglycosides, or doxycycline]). In total, 82 patients (38%) received TGC monotherapy, 42 patients (19%) underwent TGC plus doxycycline therapy, 39 patients (18%) received TGC plus ciprofloxacin therapy, and 14 patients (6%) underwent ciprofloxacin monotherapy.

### Patient outcomes according to the antibiotic(s) administered

**Fig 1:**
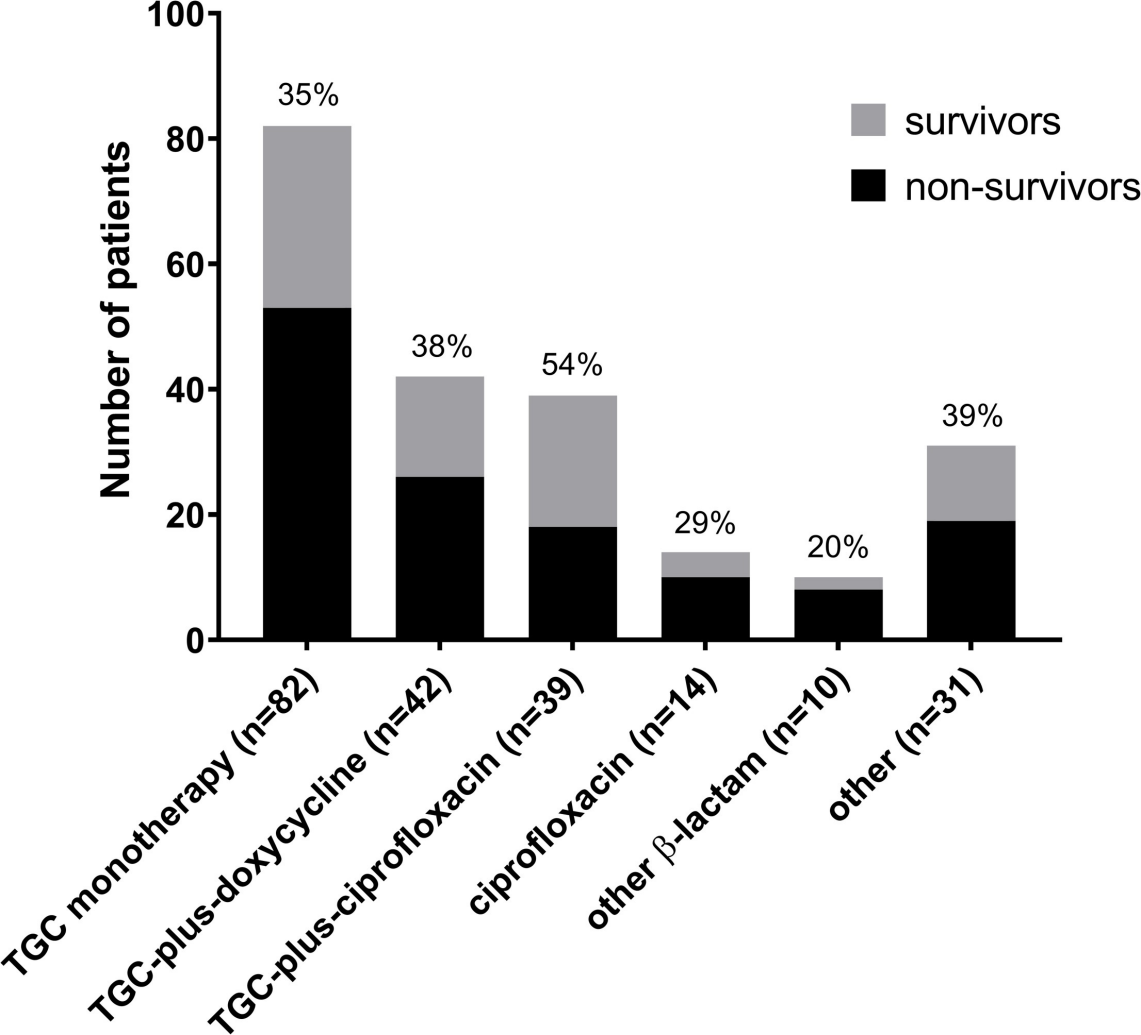
Thirty-day survival rate according to the antibiotic used in patients with *V*. *vulnificus* septicemia. Third-generation cephalosporin (TGC) refers to any of the following: cefotaxime, ceftriaxone, ceftizoxime, or cefpiramide. Other β-lactam antibiotic refers to any of the following: ampicillin, amoxicillin, ampicillin/sulbactam, or piperacillin/tazobactam. Other antibiotic refers to any of the following or any combination therapy other than TGC plus doxycycline or TGC plus ciprofloxacin: aminoglycoside (gentamicin, amikacin, or netilmicin), glycopeptide (vancomycin or teicoplanin), clindamycin, aztreonam, and a first- or second-generation cephalosporin. In this study, oral doxycycline was the only tetracycline analogue used and ciprofloxacin the only quinolone.

The survival rates at 30 days after hospital admission are presented in Fig 1. TGCs were the most frequently administered antibiotics. The TGC plus ciprofloxacin group showed the highest (54%) and the other β-lactam monotherapy group the lowest (20%) 30-day survival rate. In the ciprofloxacin monotherapy group, the 30-day survival rate was 29% (4/14) and nine patients died within 48 h. The log-rank test showed no statistical significance among six antibiotic(s) groups (*P* = 0.22). Pairwise *post hoc* analysis showed statistical significance only between TGC plus ciprofloxacin and other β-lactam (*P* = 0.02).

**Fig 2:**
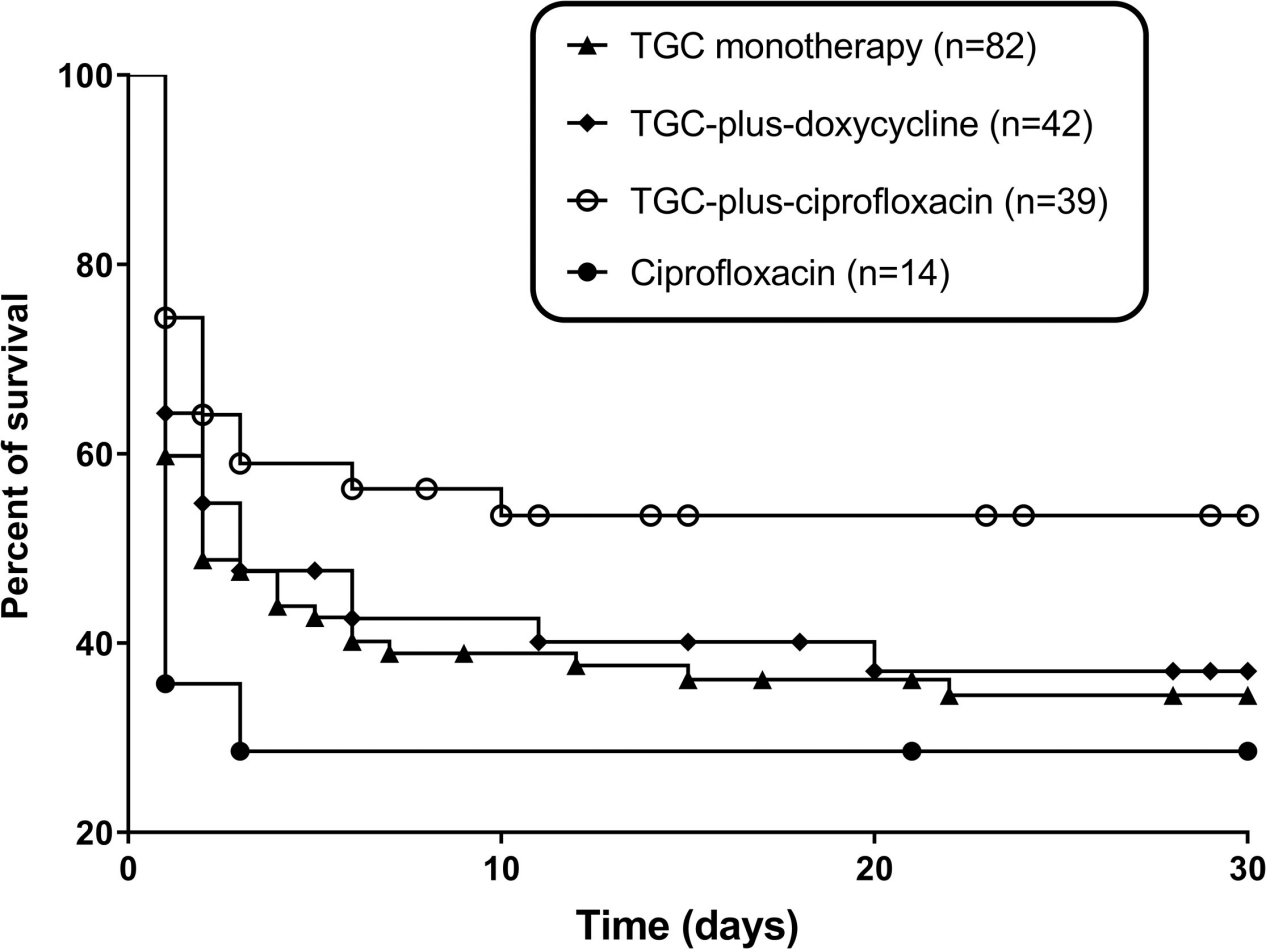
Kaplan-Meier survival curves of patients who received TGC monotherapy, TGC plus doxycycline, TGC plus ciprofloxacin, or ciprofloxacin monotherapy for the treatment of a *V*. *vulnificus* infection. A pairwise *post hoc* test showed a trend toward a higher 30-day survival rate in TGC plus ciprofloxacin than TGC monotherapy (*P* = 0.06), or ciprofloxacin monotherapy group (*P* = 0.057). There was no significant difference in survival rate between the TGC plus ciprofloxacin group and TGC plus doxycycline groups (*P* = 0.18).

A Kaplan-Meier survival analysis and log-rank test (Fig 2) showed that the TGC plus ciprofloxacin group had a trend toward a higher 30-day survival rate than the TGC monotherapy group (*P* = 0.06) or ciprofloxacin monotherapy group (*P* = 0.057). However, there was no significant difference in survival rate between the TGC plus ciprofloxacin group and TGC plus doxycycline groups (*P* = 0.18). There was no significant difference in survival rate between the TGC monotherapy and TGC plus doxycycline groups (*P* = 0.76).

The demographic characteristics of the TGC plus ciprofloxacin and TGC plus doxycycline groups are shown in Table 2. Both the proportion of patients with chronic hepatitis B and the white blood cell count were higher in the TGC plus doxycycline group. Moreover, the patients in the TGC plus ciprofloxacin group received early surgical intervention more frequently than those in the TGC plus doxycycline group.

**Table 2:** Demographic, clinical and laboratory characteristics of *V*. *vulnificus* septicemic patients who treated with TGC-plus-ciprofloxacin and TGC-plus-doxycycline.

Variables		TGC-plus-ciprofloxacin, n = 39	TGC-plus-doxycycline, n = 42	*P* value
Age (year), mean ± SD ^b^		57.2 ± 9.1	59.9 ± 10.4	0.23
Gender				
	Male ^d^	33 (85)	36 (86)	0.89
	Female	6 (15)	6 (14)	
Accompanied NSTI ^a^^,^ ^d^		35 (90)	41 (98)	0.14
	2 or more extremity involvement ^d^	17/35 (49)	28/41 (68)	0.11
Raw seafood ingestion history ^d^		33 (85)	38 (91)	0.42
Underlying disease † ^d^				
	Chronic hepatitis B	19 (49)	35 (83)	<0.001
	Liver cirrhosis	7 (18)	10 (24)	0.51
	Diabetes mellitus	7 (18)	6 (14)	0.65
	Chronic heavy alcohol drinker	13 (33)	6 (14)	0.07
APACHE II ^a^ score ^c^^,^ ^‡^		13 (9, 15)	14 (9.75, 16)	0.57
WBC ^a^ (×10^9^/L) ^c^		6.8 (3.8, 1.46)	4.7 (2.05, 8.55)	0.02
Hemoglobin (g/L) ^b^		12.4 ± 2.2	11.9 ± 2.5	0.35
Platelet (/L) ^c^		58 (41, 86)	49 (33, 79)	0.24
Creatinine (mg/dL) ^c^		1.8 (1.0, 2.5)	1.8 (1.3, 3.05)	0.29
Septic shock at admission ^d^		23 (59)	25 (56)	0.96
Early surgical intervention (within 24 h of arrival) ^d^		23 (59)	3 (7)	<0.001
Early mortality (within 48 h of admission) ^d^		8 (21)	15 (36)	0.13
30-day survival ^§^		21 (53)	16 (38)	0.18

^a^ Abbreviations: NSTI necrotizing skin and soft tissue infection, APACHE II acute physiology and chronic health evaluation II, WBC white blood cells

^b^ Continuous variables are expressed as means ± SD and were compared by the Student t test

^c^ Continuous variables are expressed as medians (IQR) and were compared by the Mann-Whitney U test

^d^ Dichotomous variables were compared by Chi-square test

† One patient might have more than 1 underlying disease

‡ All patients of TGC-plus-doxycycline and TGC-plus-ciprofloxacin included APACHE II score

§ Log rank test was used to compare 30-day survival

We analyzed demographic characteristics of the TGC plus ciprofloxacin and TGC plus doxycycline groups in 128 *V*. *vulnificus* septicemic patients who had not received any surgical treatment (Table 3). APACHE II score was higher in TGC plus doxycycline group and 30-day mortality was also higher. Therefore, we performed a propensity-score-matched analysis of the TGC plus doxycycline and TGC plus ciprofloxacin groups.

**Table 3:** Demographic, clinical and laboratory characteristics of *V*. *vulnificus* septicemic patients who had not received surgical treatment.

Variables			Not received surgical intervention, n = 128	TGC-plus-doxycycline group, n = 30	TGC-plus-ciproflxoacin group, n = 9	*P* value
Age (year), mean ± SD ^b^			57.8 ± 9.5	61.4 ± 10.1	58.4 ± 8.2	0.43
Gender						
	Male ^d^		110 (86)	24 (80)	8 (89)	1.00
	Female		18 (14)	6 (20)	1 (11)	
Accompanied NSTI ^a^^,^ ^d^			115 (90)	29 (97)	7 (78)	0.13
	2 or more extremity involvement ^d^		78/115 (68)	21/29 (72)	3/7 (43)	0.06
Raw seafood ingestion history ^d^			98 (77)	27 (90)	7 (78)	0.57
Underlying disease † ^d^						
	Chronic hepatitis B		108 (84)	25 (83)	6 (67)	0.36
	Liver cirrhosis		30 (23)	8 (27)	1 (11)	0.65
	Diabetes mellitus		19 (15)	4 (13)	2 (22)	0.61
	Chronic heavy alcohol drinker		8 (6)	4 (13)	1 (11)	1.00
APACHE II ^a^ score ^c^^,^ ^‡^			(n = 125)	(n = 30)	(n = 9)	
	Median (IQR)	14 (11, 17)	15 (12, 16)	12 (8, 14)	0.02*
WBC ^a^ (×10^9^/L) ^c^			5.8 (2.7, 11.3)	3.6 (1.8, 8.6)	14.0 (8.8, 21.1)	<0.001*
Hemoglobin (g/L) ^b^			12.2 ± 2.4	11.6 ± 2.3	12.4 ± 2.3	0.55
Platelet (/L) ^c^			52 (35, 82)	52 (36, 81)	79 (45, 109)	0.27
Creatinine (mg/dL) ^c^			1.7 (1.2, 2.7)	1.7 (1.3, 3.1)	1.8 (1.0, 3.2)	0.94
Septic shock at admission ^d^			62 (48)	19 (63)	4 (44)	0.44
Early mortality (within 48 h of admission) ^d^			61 (48)	15 (50)	2 (22)	0.25
30-day survival ^§^			30 (23)	5 (17)	5 (56)	0.10

^a^ Abbreviations: NSTI necrotizing skin and soft tissue infection, APACHE II acute physiology and chronic health evaluation II, WBC white blood cells

^b^ Continuous variables are expressed as means ± SD and were compared by the Student t test

^c^ Continuous variables are expressed as medians (IQR) and were compared by the Mann-Whitney U test

^d^ Dichotomous variables were compared by Chi-square test

† one patient might have more than 1 underlying disease

‡ All patients of TGC-plus-doxycycline and TGC-plus-ciprofloxacin included APACHE II score

§ Log rank test was used to compare 30-day survival

**Fig 3:**
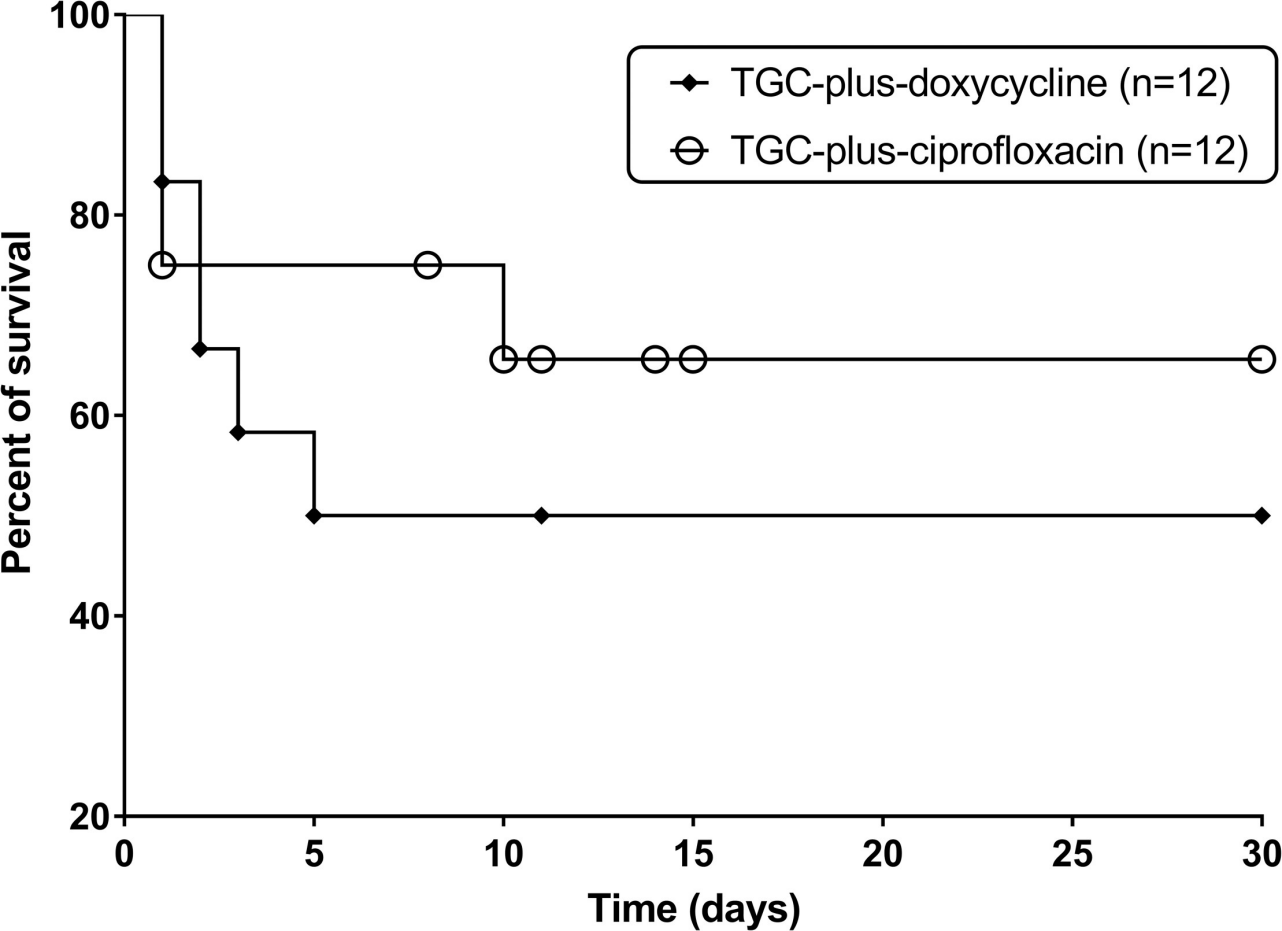
Kaplan-Meier survival curves of propensity-score-matched patients treated with a TGC plus doxycycline or TGC plus ciprofloxacin. There was no significant difference in survival between the two groups by the log-rank test (*P* = 0.46).

The 24 patients in the two groups were 1:1 propensity-score matched (Table 4). In the propensity score-matched analysis, the 30-day survival rate of the TGC plus doxycycline group was 6/12 (50.0%) and that of the TGC-plus-ciprofloxacin group was 8/12 (66.7%). The Kaplan-Meier 30-day survival curves did not differ significantly between the two groups (log-rank test, *P* = 0.46) (Fig 3).

**Table 4:** Demographic, clinical and laboratory characteristics of propensity score matched *V*. *vulnificus* septicemic patients who treated with TGC-plus-ciprofloxacin and TGC-plus-doxycycline.

Variables			TGC-plus-ciprofloxacin, n = 12	TGC-plus-doxycycline, n = 12	*P* value	
Age (year), mean ± SD ^b^			56.9 ± 7.8	61.3 ± 8.6	0.21	
Gender						
		Male ^d^	11 (92)	9 (75)	0.27	
		Female	1 (8)	3 (25)		
Accompanied NSTI ^a^^,^ ^d^			10 (83)	12 (100)	0.14	
	2 or more extremity involvement ^d^		6/10 (60)	7/12 (58)		0.68	
Raw seafood ingestion history ^d^			9 (75)	11 (92)	0.27	
Underlying disease † ^d^						
		Chronic hepatitis B	8 (67)	8 (67)	1.00	
		Liver cirrhosis	2 (17)	2 (17)	1.00	
		Diabetes mellitus	2 (17)	3 (25)	0.62	
		Chronic heavy alcohol drinker	3 (25)	3 (25)	1.00	
APACHE II ^a^ score ^c^			11.5 (7.3, 13.8)	14 (8.3, 16.8)	0.20	
WBC ^a^ (×10^9^/L) ^c^			5.0 (3.0, 11.4)	7.6 (1.6, 14.8)	0.75	
Hemoglobin (g/L) ^b^			12.3 ± 1.7	11.8 ± 2.4	0.65	
Platelet (/L) ^c^			70 (44.3, 88.5)	59 (27.8, 82.8)	0.56	
Creatinine (mg/dL) ^c^			2.1 (1.5, 3.7)	2.4 (1.4, 3.0)	0.86	
Septic shock at admission ^d^			8 (67)	8 (67)	1.00	
Early surgical intervention (within 24 h of arrival) ^d^			2 (17)	3 (25)	0.62	
Early mortality (within 48 h of admission) ^d^			1 (8)	2 (17)	0.34	
30-day survival §			8 (67)	6 (50)	0.46	

^a^ Abbreviations: NSTI necrotizing skin and soft tissue infection, APACHE II acute physiology and chronic health evaluation II, WBC white blood cells

^b^ Continuous variables are expressed as means ± SD and were compared by the Student t test

^c^ Continuous variables are expressed as medians (IQR) and were compared by the Mann-Whitney U test

^d^ Dichotomous variables were compared by Chi-square test

† one patient might have more than 1 underlying disease

§ Log rank test was used to compare 30-day survival

### Risk factors for mortality

In the multivariate analysis, septic shock at admission and an elevated APACHE II score were independent risk factors for mortality, and early surgical intervention was an independent prognostic factor for a lower mortality rate (Table 5). The adjusted odds ratio of the APACHE II score was 1.24 (*i*.*e*., the 30-day mortality increased by 24% for each increment in the APACHE II score).

**Table 5:** Risk factors for mortality in 218 patients with *V*. *vulnificus* infection.

Characteristics		Univariate analysis				Multivariate analysis			
	Value					95% CI ^a^			
	Nonfatal group n = 85		Fatal group n = 133	Odds Ratio	*P* value	Lower	Upper	Adjusted Odds Ratio	*P* value
Accompanied NSTI, 2 or more extremity involvement	42 (49)		82 (62)	1.65	0.08	0.53	2.17	1.08	0.84
Chronic hepatitis B	63 (74)		109 (82)	1.59	0.17	0.88	5.18	2.12	0.10
Septic shock at admission	28 (33)		78 (61)	3.06	<0.001*	1.34	6.41	2.93	0.01*
Early surgical intervention	28(33)		32 (24)	0.65	0.15	0.19	0.87	0.41	0.02*
APACHE II ^a^ score	11 (7, 14)		15 (12, 18))	1.24	<0.001*	1.14	1.36	1.24	<0.001*
Creatinine	1.7(1.3, 2.4)		1.9 (1.2, 2.8)	1.31	0.18	0.93	1.85	1.31	0.12

^a^ Abbreviations: APACHE II; acute physiology and chronic health evaluation II, CI; confidence interval.

* *P*<0.05

## Discussion

In this study, > 90% of patients had necrotizing skin lesions. The early mortality rate was 37% and the 30-day mortality rate was 61% in patients with septicemia and/or NSTI caused by *V*. *vulnificus*. Of the *V*. *vulnificus-*infected patients, 75% received TGC-based antibiotics. The TGC plus ciprofloxacin group had the highest 30-day survival rate (54%). In the propensity score-matched study, the 30-day survival rate of the TGC plus doxycycline group was 6/12 (50%) and that of the TGC plus ciprofloxacin group was 8/12 (67%). Therefore, TGC plus ciprofloxacin was as effective as TGC plus doxycycline. Early surgical intervention (within 24 h of admission) was an independent prognostic factor for survival.

According to US surveillance data, patients with foodborne illness have higher rates of septicemia (87% *vs*. 55%) and death (61% *vs*. 17%) than those with wound infections [30, 31]. In this study, more than 80% of the patients had a history of raw seafood ingestion and none of the patients were diagnosed with primary wound infection. It might explain the high mortality rate (> 60%).

TGC plus doxycycline combinations are standard treatments for *V*. *vulnificus* skin and soft-tissue infections according to the CDC and Infectious Diseases Society of America [23, 32]. Chen *et al* [19] reported that the case-fatality rate of ciprofloxacin with/without minocycline was not different from that of TGC plus minocycline for NSTI caused by *V*. *vulnificus*. In that study, wound infections predominated (wound infection *vs*. primary septicemia: 65% *vs*. 35%) and < 30% of the patients had hepatic disorders. Hepatic diseases could be poor prognostic factors because the elevated serum ferritin level associated with these conditions promotes the survival of *V*. *vulnificus* in whole blood [33]. In this study, the ciprofloxacin monotherapy group had a low survival rate (28.6%). The early mortality rate (64%, 9/14) of the ciprofloxacin monotherapy group was significantly higher than that of the groups treated with TGC-based regimens (21–40%). Ciprofloxacin monotherapy group showed the longer time from symptom onset to hospital visit insignificantly. An evaluation of the efficacy of ciprofloxacin monotherapy was hampered by small number and its short duration of administration, which may be insufficient to demonstrate efficacy. A previous study of the antibiotic resistance of *V*. *vulnificus* in seafood, including oysters, purchased from fish markets in Korea reported rates of resistance to cefotaxime, tetracycline, and ciprofloxacin of 11.8%, 5.9%, and 17.6%, respectively [34]. The emergence of multidrug-resistant *V*. *vulnificus* has been confirmed in several regions [5]. A study in South Carolina and Georgia of clinical and environmental *V*. *vulnificus* isolates showed that 45% of the environmental isolates were resistant to three or more classes of antibiotics and 17.3% were resistant to eight or more antibiotic agents [35]. Therefore, ciprofloxacin monotherapy may not be effective against serious *V*. *vulnificus* infections (*e*.*g*., foodborne disease).

In a mouse model of foodborne *V*. *vulnificus* septicemia, the survival rate of the ceftriaxone plus ciprofloxacin group was 100%, compared with 91% for the ceftriaxone-doxycycline group; the difference was not significant [22]. In this study, TGC monotherapy did not yield favorable outcomes, consistent with previous reports [18, 19]. Confirming the synergistic effect of a quinolone and TGC against Gram-negative pathogens (35–39), recent studies have demonstrated the efficacy of cefotaxime plus ciprofloxacin against *V*. *vulnificus in vitro* and *in vivo* [36, 37]. The interaction of quinolones with the bacterial outer membrane, with the quinolones acting as chelating agents to increase its permeability to β-lactam antibiotics, may underlie this synergistic effect [38]. The additive activity of fluoroquinolones against *V*. *vulnificus* is mediated by inhibition of the transcription of repeats-in-toxin [25]. Ciprofloxacin has low endotoxin release potential and exerts immunomodulatory effects such as altering the serum cytokine and chemokine profile [39–41]. The combination therapy with two antimicrobial agents having different targets may limit the risk of emergence of resistant mutant isolates compared to the risk resulting from monotherapy. These phenomena may explain the favorable outcomes of TGC plus ciprofloxacin therapy.

This work included the largest number of *V*. *vulnificus* septicemic patients of any single-institution study. The main limitation was that this was a 26-year retrospective analysis. Even though there was no significant change of outcome between the first and the second half of the time (35% survival in the first half, 51% survival in second half, *P* = 0.10, log rank test), the long time period could make potential for multiple confounding factors. TGC plus ciprofloxacin therapy was frequently administered in more recent years. Very diverse antibiotics in the six treatment groups were administered and individual decisions about antibiotics selection may result in selection bias. Although the internist communicated with the surgeon regarding the need for surgical intervention as soon as possible after the patient’s arrival at the emergency room, the decision to perform debridement (as well as the timing thereof) was not made based on standardized criteria. Many patients did not receive early surgical intervention due to various reasons such as host factors and reluctance of family to surgery, etc. The small portion of patients with early surgical intervention may be a confounding factor. Nevertheless, we believe that this is the actuality of the real world. We recommend temporizing surgical management according to the spread of skin lesions in patients with foodborne primary *V*. *vulnificus* septicemia with multi-extremity involvement because such patients are not clinically homogeneous. Second, various TGCs were administered and oral doxycycline was available only. Third, we could not evaluate the clinical efficacy of ciprofloxacin monotherapy due to the high early mortality rate of the patients treated with this agent. Lastly, we did not obtain a sufficient sample size to ensure adequate power to show difference. For two-sample noninferiority log-rank test by the statistical power 0.8, each 60 sample size are needed. Further research is thus warranted.

Their sporadic occurrence hampers determination of the efficacy of antimicrobials against human *V*. *vulnificus* infections. Our propensity-score-matched analysis showed no significant difference in efficacy between TGC plus ciprofloxacin and TGC plus doxycycline regimens. This is to our knowledge the first clinical report of the non-inferior efficacy of TGC plus ciprofloxacin compared with TGC plus doxycycline in patients with *V*. *vulnificus* septicemia. The results suggest that TGC plus ciprofloxacin should also be a CDC recommended treatment for patients with a *V*. *vulnificus* septicemia.
